# Enhanced intelligent approach for determination of crude oil viscosity at reservoir conditions

**DOI:** 10.1038/s41598-023-28770-2

**Published:** 2023-01-30

**Authors:** Kiana Peiro Ahmady Langeroudy, Parsa Kharazi Esfahani, Mohammad Reza Khorsand Movaghar

**Affiliations:** Department of Petroleum Engineering, Amirkabir University of Technology (Tehran Polytechnic), 424 Hafez Avenue, Box 15875-4413, Tehran, 1591634311 Iran

**Keywords:** Crude oil, Chemical engineering, Scientific data

## Abstract

Oil viscosity plays a prominent role in all areas of petroleum engineering, such as simulating reservoirs, predicting production rate, evaluating oil well performance, and even planning for thermal enhanced oil recovery (EOR) that involves fluid flow calculations. Experimental methods of determining oil viscosity, such as the rotational viscometer, are more accurate than other methods. The compositional method can also properly estimate oil viscosity. However, the composition of oil should be determined experimentally, which is costly and time-consuming. Therefore, the occasional inaccessibility of experimental data may make it inevitable to look for convenient methods for fast and accurate prediction of oil viscosity. Hence, in this study, the error in viscosity prediction has been minimized by taking into account the amount of dissolved gas in oil (solution gas–oil ratio: R_s_) as a representative of oil composition along with other conventional black oil features including temperature, pressure, and API gravity by employing recently developed machine learning methods based on the gradient boosting decision tree (GBDT): extreme gradient boosting (XGBoost), CatBoost, and GradientBoosting. Moreover, the advantage of the proposed method lies in its independence to input viscosity data in each pressure region/stage. The results were then compared with well-known correlations and machine-learning methods employing the black oil approach applying least square support vector machine (LSSVM) and compositional approach implementing decision trees (DTs). XGBoost is offered as the best method with its greater precision and lower error. It provides an overall average absolute relative deviation (AARD) of 1.968% which has reduced the error of the compositional method by half and the black oil method (saturated region) by five times. This shows the proper viscosity prediction and corroborates the applied method's performance.

## Introduction

As a measure of fluid resistance to flow, viscosity is found in any equation dealing with fluid flow, including equations of flow in porous media^1–3^. The oil viscosity is an essential parameter employed in reservoir performance evaluation and simulation, surface facility design, and identification of the optimal production scenario in a reservoir^4–8^. It is also crucial in tertiary recovery techniques, e.g., thermal enhanced oil recovery (EOR), affecting the oil viscosity directly^9,10^. Therefore, it is essential to accurately calculate the viscosity of crude oil using advanced and accurate techniques in petroleum engineering.

The viscosity of crude oil is often measured experimentally. Oil samples can be produced under subsurface/underground (reservoir) conditions or collected at surface conditions. In the latter, they are produced through the recombination of the gas and fluid from the separators. Experimental techniques are expensive and, in most cases, time-consuming. Hence, it is necessary to perceive numerical methods in order to accurately predict the viscosity of crude oil at different pressures and temperatures, particularly when pressure–volume–temperature (PVT) experimental data are unavailable.

Based on input parameters, there are two types of equations for estimating oil viscosity^7^. The first, known as the black oil method, uses conventional oil field data such as temperature, reservoir pressure, saturation pressure, solution gas-oil ratio (R_s_), and API gravity. However, for proper calculation in the compositional material balance, the reservoir oil and gas viscosity should be accurately estimated based on their components^11^. Therefore, the second type has been developed based on the effect of oil composition (the type and fraction of components). The input parameters of the compositional method include oil composition, critical temperature, molecular mass, acentric factor, normal boiling point, and pour point^7,12–14^. It is worth noting that the supplementary file-comparison with the preexisting models provides well-known equations for black oil and compositional material balance models.

At the same time, the pressure reduction in the sub-bubble-point region along with solution gas reduction in oil adds to the weight and viscosity of the oil. In other words, the oil composition below the bubble pressure changes upon a decreased pressure, altering the oil viscosity. Therefore, there is a need to apply another pressure-based type division to current methods (computational approaches and correlations) as a classifier to categorize oil viscosity into three regions: (1) dead oil, (2) saturated oil, and (3) undersaturated oil. The first step in applying these equations is calculating the dead oil viscosity. Hence, an accurate calculation of dead oil viscosity must be conducted prior to the next steps (i.e., viscosity at the bubble point and viscosity at the reservoir pressure and temperature)^1,15^.

Despite the simple use of empirical equations to predict viscosity, each is developed based on a particular dataset (input parameters) and regions. So, deployment of them would be inaccurate for other datasets and regions. In other words, a given empirical equation cannot be generalized. Hemmati-Sarapardeh et al.^16^ listed common empirical equations for oil viscosity prediction with the datasets and regions used in their development.

Accordingly, soft computing techniques (artificial intelligence (AI) and machine learning (ML)) are developed based on optimization algorithms as efficient methods in order to predict viscosity^16–25^ accurately. These techniques have mainly been developed based on the black oil model.

Using an artificial neural network (ANN) code in MATLAB, Lashkenari et al.^17^ provided a model aiming to estimate the viscosity of Iranian (reservoir) oil samples. Input parameters including temperature, pressure, solution gas-oil ratio (R_s_), and API gravity, at three different regions relative to the bubble-point pressure were applied in the prediction procedure of the viscosity. It was concluded through considering previous studies that, the ANN model has higher precision as well as better efficiency.

In another attempt for the same regions and the same input parameters, Hemmati-Sarapardeh et al.^19^ applied the least squares support vector machine (LSSVM) method. In their study, API gravity, temperature, pressure, and most importantly viscosity (experimental) were defined as input parameters. Predictions showed that the LSSVM model performed notably better than the well-known correlations with acceptable agreement compared to the experimental data.

In another study, through the application of coupled simulated annealing technique in the optimization of least square support vector machine modelling, Hemmati-Sarapardeh et al.^16^ attempted to improve the results merely for the saturated region.

In an attempt of an obtaining efficient polynomial correlation for estimating oil viscosity, Ghorbani et al.^18^ applied a hybrid group method of data handling (GMDH) artificial neural network, optimized with genetic algorithm (GA). Hence, A large data set of Iranian crude oils employing multiple variables, including API gravity, (saturation) pressure, and reservoir temperature was used. Their results indicated that these models can be considered fine estimations.

Using various soft computing techniques purposefully decision tree (DTs) and random forest (RF), Talebkeikhah et al.^20^ developed a compositional model for undersaturated, saturated, and dead oil regions. It is noteworthy to mention that, in their model, the molecular weight of C_12_^+^ and the molar fractions of C_1_ − C_11_ were added as input parameters, besides the black oil parameters. They concluded that DTs outperforms the available approaches.

In a multiphase reservoir oil system, and through the application of machine learning approaches Shao et al.^21^ developed three viscosity prediction models. Input data, including gas-oil and water–oil molar ratios, reservoir pressure, and reservoir temperature were used. It was concluded that random forest (RF) model performance had considerable accuracy in estimating the viscosity of existing phases in the reservoir. Moreover, sensitivity analysis indicated that the gas-oil molar ratio is the determining factor in affecting the viscosity of each phase, in a multiphase reservoir.

In an attempt of predicting viscosity, Aladwani and Elsharkawy^22^ implemented three supervised machine learning regression (SMLR) models. The density parameter was their opted additional input parameter in addition to the common black oil parameters (API, temperature, and pressure). It should be noted that while the density parameter is always considered as an input parameter in compositional modelling, the inclusion of the density parameter as black oil model input parameter was a contrast in their study. Finally, they concluded that the Gaussian process regression (GPR) and the regression ensembles tree (RET) had the best performance.

It is noteworthy to mention the fact that, considering the dead oil viscosity as an input feature in prediction of the saturated oil viscosity, numerous studies using machine learning and artificial intelligence approaches have already been performed for the precise estimation of this parameter^13,26–28^.

This study accurately estimates crude oil viscosity under reservoir conditions using ensembled machine learning methods through only black oil parameters and without costly oil compositional analysis. In this communication, a large databank of Iranian oil reservoirs, measured using a Rolling Ball viscometer (Ruska, series 1602) is applied in developing the new models (Refer to supplementary file-materials and methods). This dataset covers a wide range of Iranian oil reservoirs’ PVT data, and it can be inferred that; the developed models could be reliable for the prediction of other Iranian oil reservoirs’ viscosity. For this purpose, based on 1368 Iranian oil reservoir data points, three new models are proposed in an attempt of predicting the under-saturated, saturated, and dead oil viscosity regions. Therefore, three rigorous soft computing schemes were implemented, namely extreme gradient boosting (XGBoost), CatBoost, and GradientBoosting. Input parameters including pressure, temperature, API gravity, and solution gas-oil ratio (R_s_) are employed. A quantitative and qualitative analysis of the model is carried out aiming to establish the adequacy and accuracy of the model. The performance of new models is evaluated in comparison with the earlier ML models under black oil and compositional approaches through statistical error analysis. The novelty of the proposed method lies in its independence to input viscosity data. This indicates that neither numerically calculated viscosity data using soft computing techniques nor empirical viscosity data (experimental/available data) are used to predict viscosity at higher pressures.

The remainder of the manuscript is organized as follows: “Model” section highlights the basics and algorithms of each implemented soft computing technique in the study. “Results and discussion” section, description of the methodology, model development, as well as results & discussion are given. Lastly, in “Conclusion” section, the main findings are summarized.

## Model

In the present study, the ensemble type of machine learning method, an emerging line of research, is employed. An ensemble classifier integrates multiple classifiers to increase robustness and represent an improved version of classification performance from any of the constituent classifiers. Additionally, this technique, in comparison to a single classifier technique, is more resilient to noise^29^. The following ensemble methods are used in this study: GradientBoosting, CatBoost, and XGBoost machines that all these methods are developed using a gradient boosting decision tree (GBDT)^30,31^.

### GradientBoosting^32^

The boosting technique focuses on iteration and reconsideration of the errors in each step to develop a strong learner by integrating multiple weak learners. The data selected to train the model can be defined by assuming $x=\{{x}_{1},{x}_{2}, \dots , {x}_{n}\}$ as the features of interest and y as the target data. In general, this method aims to find the approximate value of $\widetilde{F}\left(x\right)$ for *F(x)* according to this condition:

1 $$\widetilde{F}\left(x\right)=\mathit{arg}\underset{F\left(x\right)}{\mathit{min}}{L}_{y,x}\left(y,F\left(x\right)\right)$$

where ${L}_{y,x}\left(y,F\left(x\right)\right)$ is the cost function and $\mathrm{arg}\underset{F\left(x\right)}{\mathrm{min}}{L}_{y,x}\left(y,F\left(x\right)\right)$ is the value of *F(x)* for which ${L}_{y,x}\left(y,F\left(x\right)\right)$ achieves its minimum. The cost function improves the parameter prediction accuracy by reaching the smallest value. Each of the weak learners tries to improve and reduce the previous weak learner’s error. In the end, the desired regression tree function (i.e.,$h({x}_{i};a)$) for parameter *a* representing a weak learner should be obtained. Each decision tree is then matched and adapted to its determined slope. ${F}_{m}\left(x\right)$ is updated in the final step based on each iteration done^33^. For more detailed information please refer to the supplementary file-GradientBoosting.

### CatBoost^34,35^

CatBoost is a relatively novel GBDT based method. A feature of GBDT is that it operates properly on datasets with numerical features. However, some datasets may include string features (e.g., gender or country) rather than merely numerical features. Hence, these features might be of great importance and have substantial effects on the accuracy of our final prediction, it is impossible to ignore or remove them. Therefore, it is customary to convert categorical (string) features into numerical features before a dataset is trained. Unlike some other GBDT based methods, an outstanding advantage of the CatBoost model is that it can handle categorical features in the training process.

As defined earlier, categorical features are non-numerical. So, for using them in our model, we must first convert them into numbers and then begin the training process of the model. For more information about these conversion methods and Catboost solution for possible problems^36^ during this proccess, please refer to the supplementary file-CatBoost.

### XGBoost^37^

The extreme gradient boosting (XGBoost) algorithm, designed and introduced by Chen et al.^38^, is among the modern machine learning methods based on the gradient boosting decision tree. This algorithm aims to approximate the estimated value to the real value as much as possible by creating a large number of trees (e.g., *k*) in order to minimize errors and maximize adaptability. This algorithm integrates weak learners to create a strong learner. However, weak learners are created through residual fitting in this algorithm^39,40^. XGBoost model extends the cost function of the first-order Taylor and presents the second-order derivative information to make the model converge faster when the model is learning. Due to adding a regularization section to the cost function, the XGBoost algorithm prevents complexity and reduces the risk of overfitting. For more information about the general process of the XGBoost algorithm please refer to the supplementary file-XGBoost.

**Figure 1:**
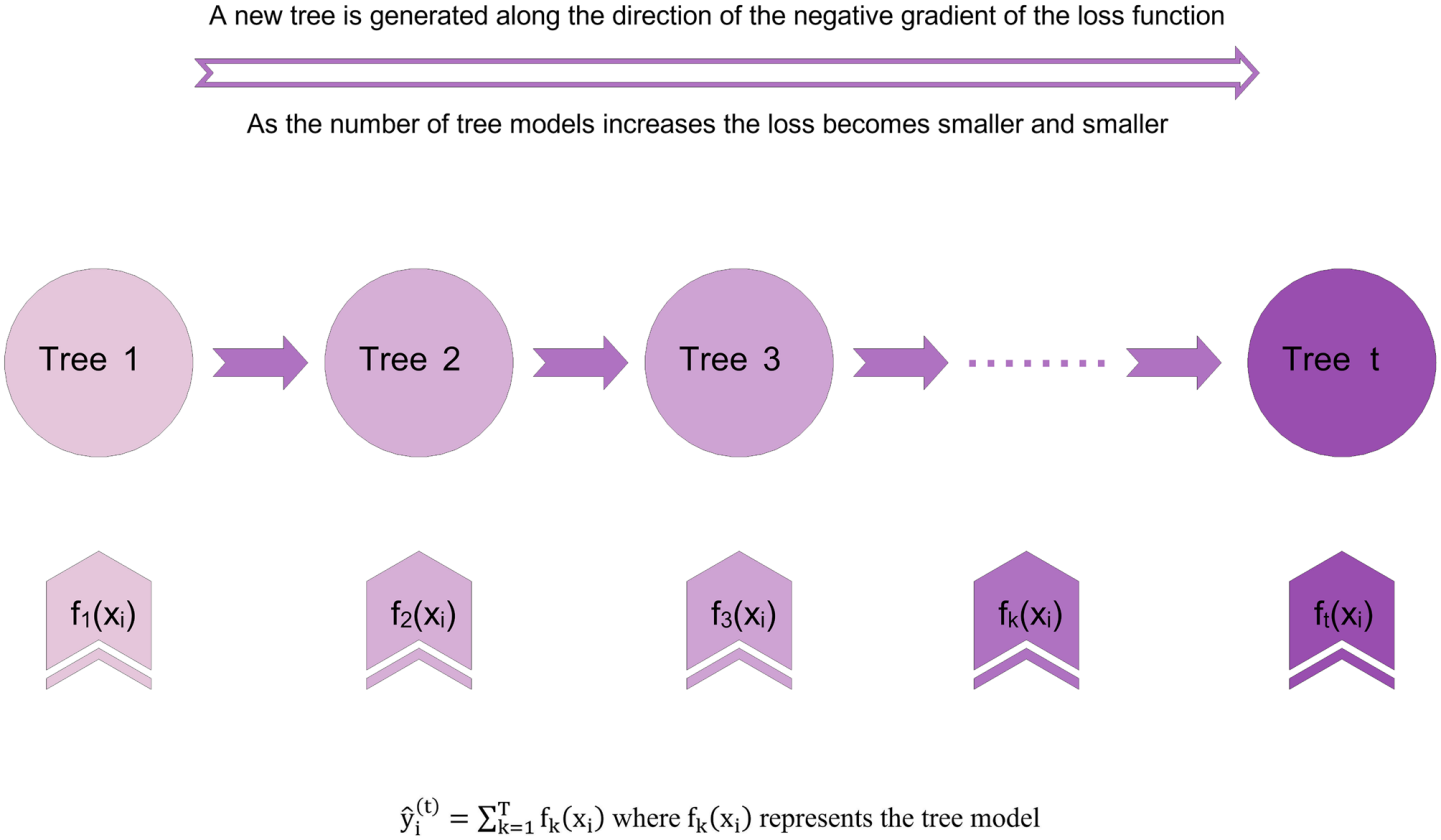
Schematic of XGBoost algorithm.

Figure 1 demonstrates the proposed algorithm structure for a simpler and more tangible understanding^41^.

## Results and discussion

### Model development

The studied databank includes experimental viscosity measurements at various pressures using a rolling-ball viscometer (Ruska, Series 1602). The experimental pressure ranged substantially above and below the bubble point of each sample (the supplementary file-materials and methods provides additional complementary describing the measurement procedure using the aforementioned tools and methods). Accordingly, 1368 experimental data were collected, fully describing the Iranian crude oil samples. These data were employed to develop efficient models for predicting viscosity more accurately. The input features for each sample were pressure, temperature, API gravity, and solution gas-oil ratio (R_s_).

**Figure 2:**
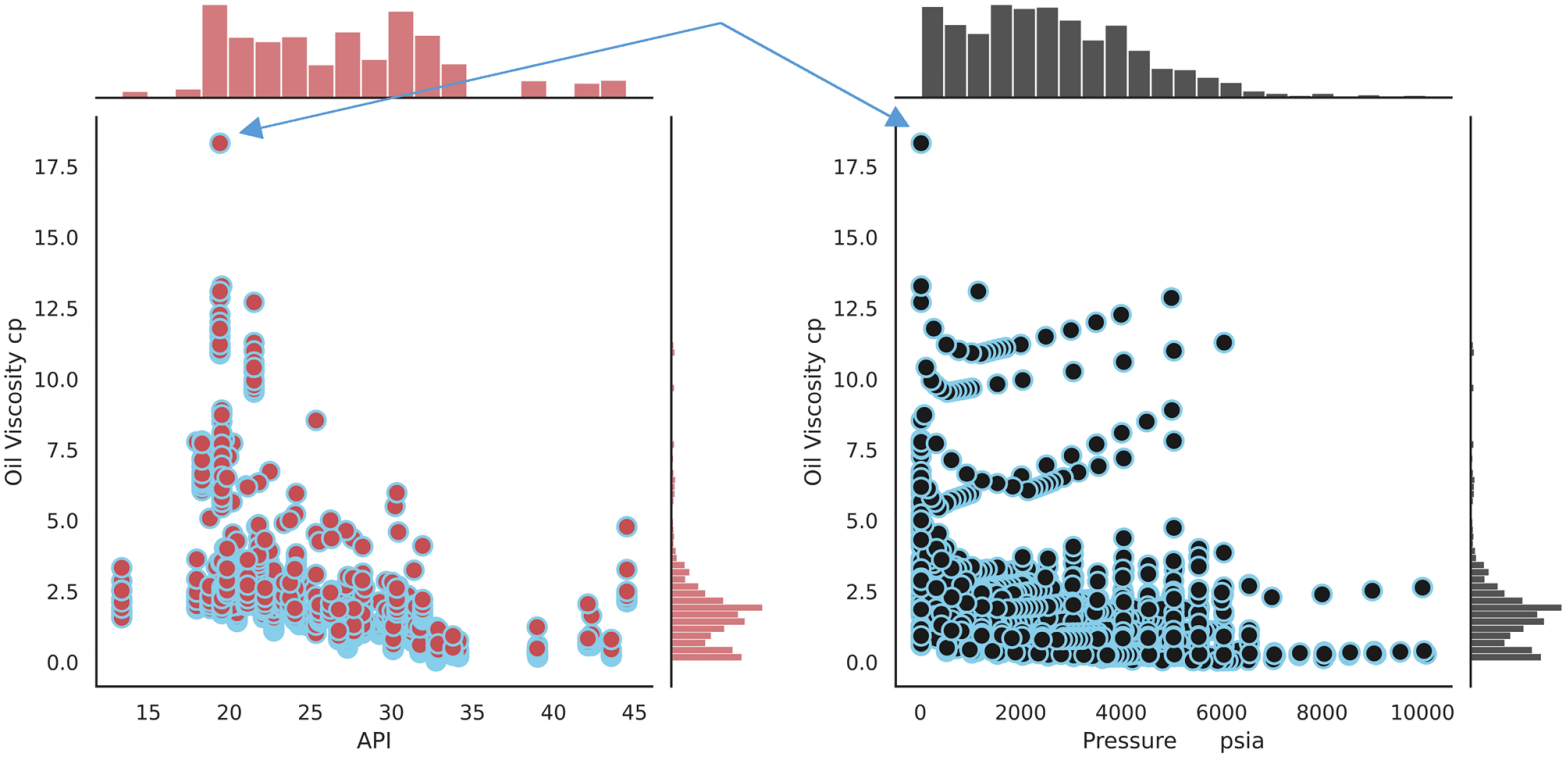
Data joint plots to check the outliers.
- This dataset consists of no missing data values.
- This dataset includes no string features and all of the features are numerical. Therefore, there is no need for using any type of encoding.
- Rescaling or normalization is an important part of preprocessing and plays an important role in model accuracy. It should be noted that tree-based models can do it by themselves, therefore there is no need to implement the rescaling process separately.

In this study, five steps are used for data preprocessing which can be given as follows:

a) data duplication, b) noise and outliers, c) missing data, d) encoding, e) rescaling features.

- Using the same data for both training and test might lead to inaccurate prediction in the process, therefore data duplication was checked in the first step.
- Checking outliers are the second step of data preprocessing. For this purpose, two joint plots are applied to analyze the points that have the potential of being outliers.
  By analyzing these Fig. 2. it can be concluded that the indicated point in both subfigures can be assumed to be an outlier. Therefore, it was decided that this point should be removed from the dataset.

Table 1 summarizes data employed for model development and the range of experimental viscosity. It is noteworthy that the experimental databank was randomly divided into two sub-groups: the first group, including 80% of experimental data, was used for training models, and the second group, including the remaining 20%, was used to measure the efficiency and reliability of models relative to the blind cases. The method mentioned above for data allocation often produces desirable and reliable results.

**Table 1:** Statistical ranges and parameters related to inputs/outputs employed for developing models.

No	Parameters	Unit	Count	Mean	Std	Min	25%	50%	75%	Max
1	Pressure	Psi	1368	2675.665	1824.740	14.700	1303.250	2442.000	3933.250	10,072.000
2	Temperature	F	1368	218.410	40.202	110.000	190.000	220.000	250.000	290.000
3	Solution GOR(R_s_)	SCF/STB	1368	641.155	572.991	0.000	334.517	478.831	772.248	4499.164
4	API	–	1368	26.913	6.481	13.350	21.520	26.740	31.000	44.520
5	Oil viscosity	C.P	1368	2.028	2.049	0.044	0.878	1.657	2.275	18.322

In this study, the grid search algorithm is used to optimize the model hyperparameters. This algorithm proposed by GridSearchCV creates candidates from a grid of hyperparameters values that could be specified then. The GridSearchCV instance uses the usual estimator/predictor API: when fitting it on a dataset all the combinations of hyperparameter values that can happen are considered and the outputs are the best hyperparameters that significantly affect the model’s final evaluation. It should be noted that, the estimator/predictor API provides methods to train the model, to judge the model accuracy^42^.

The result of hyperparameters is presented as control parameters in Table 2 for each modeling technique used in this study.

**Table 2:** Control parameters used for the development and application of soft computing techniques.

	Parameters	Value
GradientBoosting	n-estimators	45
	Max depth	7
	Learning rate	0.10
	Subsample	1
	Alpha	0.90
	Min samples split	2
XGBoost	n-estimators	99
	Max depth	9
	Learning rate	0.07
	Subsample	0.75
	Gamma	0
	Col sample by tree	1
CatBoost	Depth	8
	Learning rate	0.07
	Iterations	700
	Best model min trees	1
	Bootstrap type	MVS
	Leaf estimation method	Newton

### Performance evaluation

Statistical and graphical criteria were used to evaluate the efficiency of the proposed algorithms and models. The statistical indices used for this purpose are:

1. Average Absolute Relative Deviation (AARD).
  2 $$AARD(\%)=\frac{1}{N}\sum_{i=1}^{N}\left|\frac{{O}_{iexp}-{O}_{ipred}}{{O}_{iexp}}\right|\times 100$$
2. Coefficient of Determination (R^2^).
  3 $${R}^{2}=1-\frac{{\sum }_{i=1}^{N}{\left({O}_{iexp}-{O}_{ipred}\right)}^{2}}{{\sum }_{i=1}^{N}{\left({O}_{ipred}-\overline{O }\right)}^{2}}$$
3. Root Mean Square Error (RMSE).
  4 $$RMSE=\sqrt{\frac{1}{N-1}\sum_{i=1}^{N}{\left(\frac{{O}_{iexp}-{O}_{ipred}}{{O}_{iexp}}\right)}^{2}}$$

**Figure 3:**
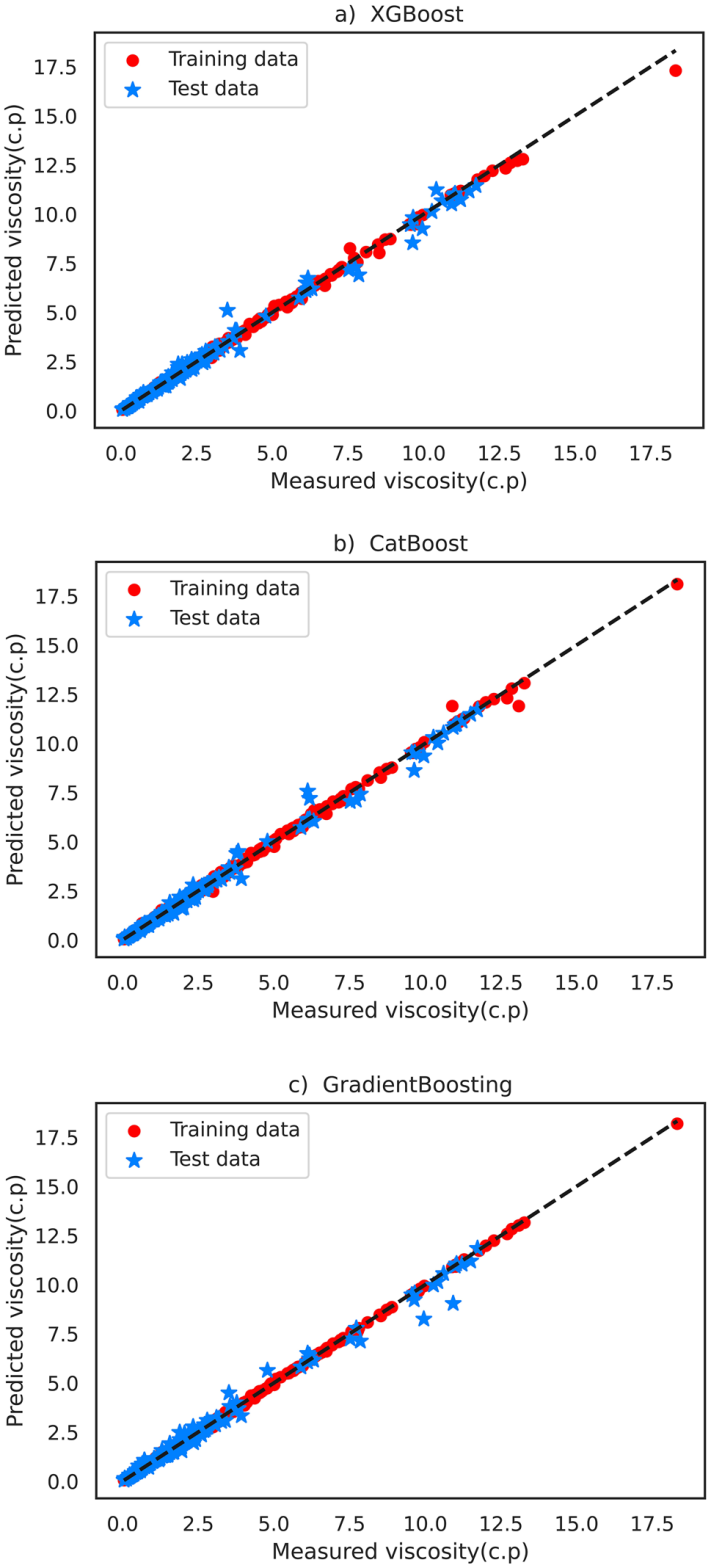
Cross plots of the implemented models: (**a**) XGBoost, (**b**) CatBoost, and (**c**) GradientBoosting.

In Eqs. (2), (3) and (4) ${O}_{i}$ represents the output (viscosity) and exp and pred denote the actual and estimated viscosity values, respectively. In addition, $\overline{O }$ is the mean of outputs, and N is the number of data points. In addition to statistical analysis, graphical evaluations were also carried out to visually show the models' capability and efficiency in accurately predicting viscosity. In this evaluation method, cross-plots are drawn to present and analyze the distribution of predictions nearby the straight line X = Y (ideal model). Figure 3 illustrates the cross-plots describing the results of the aforementioned soft computing techniques for viscosity prediction. This figure shows a uniform distribution of predictions around the slope line in XGBoost, CatBoost, and GradientBoosting models, demonstrating the efficiency of these models in properly predicting viscosity. Comparing these models reveal that the XGBoost model exhibited perfect behavior without any considerable deviation around the X = Y line, outperforming the other two models.

Some statistical indices were also reported in Table 3 for further analysis of the models. According to the results, the XGBoost model outperformed other models, with an AARD and a coefficient of determination of 1.968% and 0.9976, respectively. The same statistical indices were then employed to compare the XGBoost model with other models proposed in previous studies. The better performance of the XGBoost model can be attributed to the improvement and development of the Gradient Boosting Decision Tree (GBDT) technique in three main aspects. First, traditional GBDT uses the first-order Taylor expansion, whereas XGBoost uses the second-order Taylor expansion with the first and second orders as improved residuals. Therefore, the XGBoost model has a wider range of applications. Second, XGBoost adds a regularization term to the objective function to regulate the model's complexity. This term can reduce variance and the likelihood of training an overfitted model. Finally, XGBoost uses the random forest column sampling method to further reduce the likelihood of overfitting. XGBoost has demonstrated a great learning performance and training speed^41^.

**Table 3:** Statistical indices used for describing the performance of proposed models.

Models	Train			Test			Overall		
	RMSE	R^2^	AARD (%)	RMSE	R^2^	AARD (%)	RMSE	R^2^	AARD (%)
GradientBoosting	0.071	0.999	3.266	0.235	0.988	5.929	0.103	0.996	3.798
CatBoost	0.069	0.999	2.246	0.181	0.993	4.380	0.091	0.998	2.672
XGBoost	0.063	0.999	1.394	0.192	0.993	4.264	0.088	0.998	1.968

To show the robustness of the model we also provide a tenfold cross-validation that is performed on the training dataset. In k-fold cross-validation, the training set is divided into *k* subsets then a model is trained with k − 1 folds and the resulting model is validated on the remaining part of the data. The performance measure reported by *k*-fold cross-validation is then the average of the values computed for each fold. We reached a 95.24% R^2^-score for the average of our tenfold cross-validation. According to the obtained value of the R^2^-score, it can be concluded that the XGBoost model has a fairly high performance not only for the 20% data that we used for the test but also for the whole dataset that we used for training.

**Figure 4:**
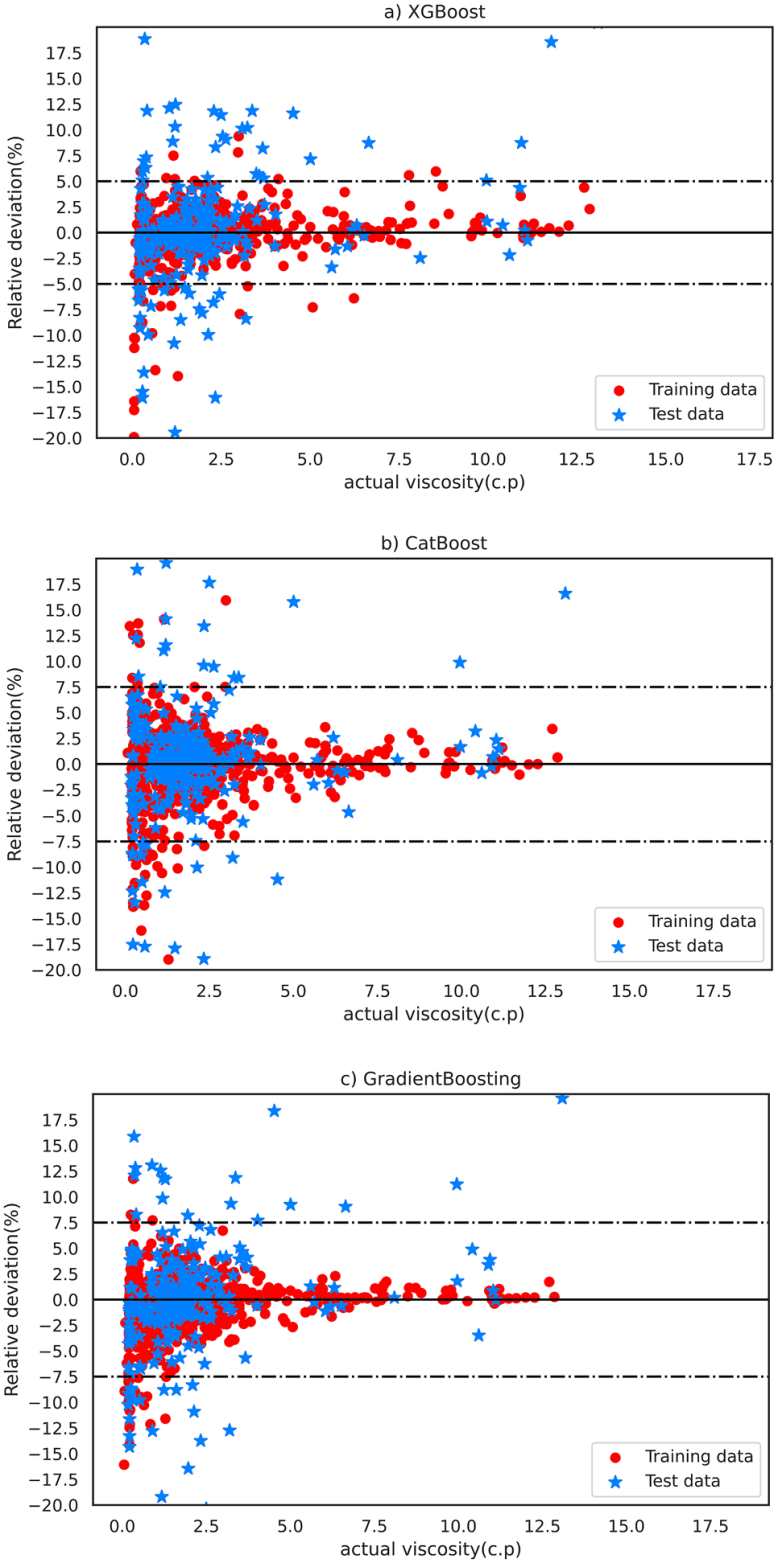
Relative deviation (%) of estimated viscosity values using the (**a**) XGBoost, (**b**) CatBoost, and (**c**) GradientBoosting model for test and train data points.

For better evaluation of the models’ performances, the relative deviation of each model’s predictions compared with the actual viscosity for test and train data is depicted in Fig. 4. As shown, the XGBoost model estimated most data with an absolute relative deviation of less than 5%, confirming the accuracy and efficiency of this model.

### Comparison of the XGBoost model with previously developed approaches

After it was shown that the XGBoost model outperformed other machine learning models, its capability and application in predicting viscosity for different pressure zones (undersaturated, saturated, and dead oil) were compared with other available approaches. Hemmati-Sarapardeh et al. introduced two approaches based on machine learning and the division of input parameters into black oil^19^ and compositional^20^ methods. The approaches were then demonstrated to outperform earlier methods and equations (supplementary file-comparison with the preexisting models).

Since 2020 till now, 326 data points are collected and added to the existing data bank. Therefore, the current study is performed based on 1368 data points. It should be noted that for a fair comparison between XGBoost and Hemmati-Sarapardeh's^19,20^ studies, the aforementioned 326 data points are excluded from the current data bank, and as a consequence, the remained 1042 data points, the same as Hemmati-Sarapardeh's^19,20^ studies are considered. In the following, the results will be reviewed and compared to the results of Hemmati-Sarapardeh's^19,20^ studies.

#### Comparison with black oil study

The black oil method input parameters of the^19^ study includes API gravity, temperature, pressure, and most importantly viscosity (experimental). The viscosity obtained in each step is used along with the other inputs to predict the viscosity in the next step. For example, dead oil viscosity is used to calculate oil viscosity at or below the bubble point, and the bubble-point viscosity is employed as an input to calculate oil viscosity at pressures above the bubble point.

Considering the fact that the oil viscosity estimation/prediction was the tangible outcome of this study, excluding viscosity from the input parameters would be reasonable. Therefore, viscosity is replaced with R_s_ in the input parameters (as mentioned in Table 1). Table 4 compares the XGBoost model (this study) with the LSSVM model proposed by^19^ for deal oil, saturated oil, and undersaturated oil regions. XGBoost outperformed the LSSVM approach, particularly in the saturated oil region. It is worth noting that, the most considerable curvature in the viscosity vs. pressure diagram is obtained in the saturated oil region, which is predicted by the XGBoost model with the lowest error.

**Table 4:** Performance of the XGBoost model in comparison with the LSSVM model.

	Models	Train			Test			Overall		
		RMSE (cP)	R^2^	AARD (%)	RMSE (cP)	R^2^	AARD (%)	RMSE (cP)	R^2^	AARD (%)
Under saturated	XGBoost^a^	0.040	0.999	0.576	0.048	0.999	1.194	0.042	0.999	0.699
	LSSVM^b^	0.030	0.999	1.500	0.040	0.999	1.400	0.040	0.999	1.400
Saturated	XGBoost^a^	0.029	0.998	2.058	0.083	0.981	5.416	0.040	0.994	2.730
	LSSVM^b^	0.310	0.988	13.500	0.770	0.838	13.200	0.380	0.979	13.480
Dead oil	XGBoost^a^	0.632	0.928	7.018	0.748	0.867	12.542	0.431	0.931	7.982
	LSSVM^b^	1.780	0.959	21.300	1.650	0.914	19.700	1.820	0.955	21.200

^a^XGBoost model (This Study).

^b^LSSVM model^19^.

#### Comparison with compositional study

The compositional model of^20^ used sixteen components of oil (methane to C_11_ and Non-hydrocarbons), C_12_^+^ molecular weight, temperature, pressure, and most importantly, viscosity (computed/predicted in each step) as input parameters. The viscosity estimated in each step was used along with the other inputs to predict oil viscosity in the next step (similar to the black oil model calculation approach).

As mentioned, the inputs of the XGBoost model include API gravity, temperature, pressure, and R_s_. Table 5 compares XGBoost and the DTs model of^20^ in the dead oil, saturated oil, and undersaturated oil regions. It can be observed that XGBoost outperformed the DTs model, except in the dead oil region, reducing the error by approximately 1.5%. It is noteworthy to emphasize that, XGBoost uses fewer input parameters than the DTs model of^20^ (4 versus 21), yielding more accurate estimates in a shorter time at a lower cost (independently of oil composition analysis), without using viscosity estimations in the previous step.

**Table 5:** Performance of the XGBoost model in comparison with the DTs model.

	Models	Train			Test			Overall		
		RMSE (Pa.s.)	R^2^	AARD (%)	RMSE (Pa.s.)	R^2^	AARD (%)	RMSE (Pa.s.)	R^2^	AARD (%)
Under saturated	XGBoost^a^	4.038E−5	0.999	0.576	4.830E−5	0.999	1.194	4.198E−5	0.999	0.699
	DTs	NR	NR	NR	NR	NR	NR	1.000E−4	0.999	2.255
Saturated	XGBoost^a^	2.956E−5	0.998	2.058	8.305E−5	0.981	5.416	4.026E−50	0.994	2.730
	DTs^b^	NR	NR	NR	NR	NR	NR	1.000E−4	0.996	4.485
Dead oil	XGBoost^a^	6.320E−4	0.928	7.018	7.481E−4	0.867	12.542	4.315E−4	0.931	7.982
	DTs^b^	NR	NR	NR	NR	NR	NR	4.000E−5	0.992	6.524
All data	XGBoost^a^	2.525E−5	0.998	1.212	5.437E−5	0.992	2.728	3.107E−5	0.997	1.515
	DTs^b^	1.000E−4	0.997	2.688	1.000E−4	0.994	6.148	1.000E−4	0.997	3.379

^a^XGBoost model (This Study).

^b^DTs model^20^.

*NR* Not Reported.

Investigating viscosity vs. pressure curves indicates insignificant variations of oil viscosity above the bubble point. This observation is related to the fact that the composition remains unchanged above the bubble point, and oil viscosity is only a function of expansion, like the other liquids. However, the fraction of dissolved gases in oil decreases at pressures below the bubble point (oil and gas phases are in an equilibrium phase within the reservoir), resulting in a notable increase in the viscosity. In fact, the oil composition influences its viscosity below the bubble point, and a reduction in the dissolved gases raises the oil viscosity. In other words, a change in the dissolved gas fraction represents a change in the oil composition. Consequently, the inclusion of R_s_ into the set of input parameters in the XGBoost model based on the black oil approach yielded more accurate oil viscosity estimates than earlier compositional work^20^.

Moreover, asphaltene and resin affect the viscosity which could be considered in two parts. Firstly, the direct effect of the content of these components on the bulk properties of the oil (crude or live), e.g. density and (dead oil) viscosity (thermodynamic effect). For this part, even small content of asphaltene will lead to a considerable increase in viscosity and density while for resins much higher content can lead to higher viscosity and density. The second perspective is the precipitation of these fractions into the new distinct phase that results in a drastic increase in oil viscosity, the kinetic and hydrodynamic effects. It should be noted that resins increase the solubility of asphaltenes in oil and also contribute to the dispersion of asphaltene. Therefore, the amount of resin and asphaltene affect the amount of viscosity and density directly when they are soluble in the oil. However, as suspensions and colloids, they should be correlated with other distinct methods and approaches^43^.

Next, in order to improve the reliability of comparison, the reason for the superiority of the XGBoost model to other decision tree methods should be discussed. The XGBoost model is based on the GBDT technique, in which the boosting strategy is adopted to integrate several (i.e., n) decision trees through a powerful and efficient technique. The number of trees depends on the number and type of data; hence, a strong learner is created. However, the DTs model is among the machine learning approaches that employ a tree-like framework to handle a wide range of input types and find the appropriate path for the prediction of results. At the same time, the DTs model can sometimes be vulnerable to overfitting. It is also sensitive to the noise in data. The concurrent use and integration of several DTs models can compensate for the lack of accuracy in each model and reduce the overall error. As a result of this procedure, the models like XGBoost that have been developed through the GBDT can outperform the DTs models in estimating the outputs^23^.

### Samples

**Figure 5:**
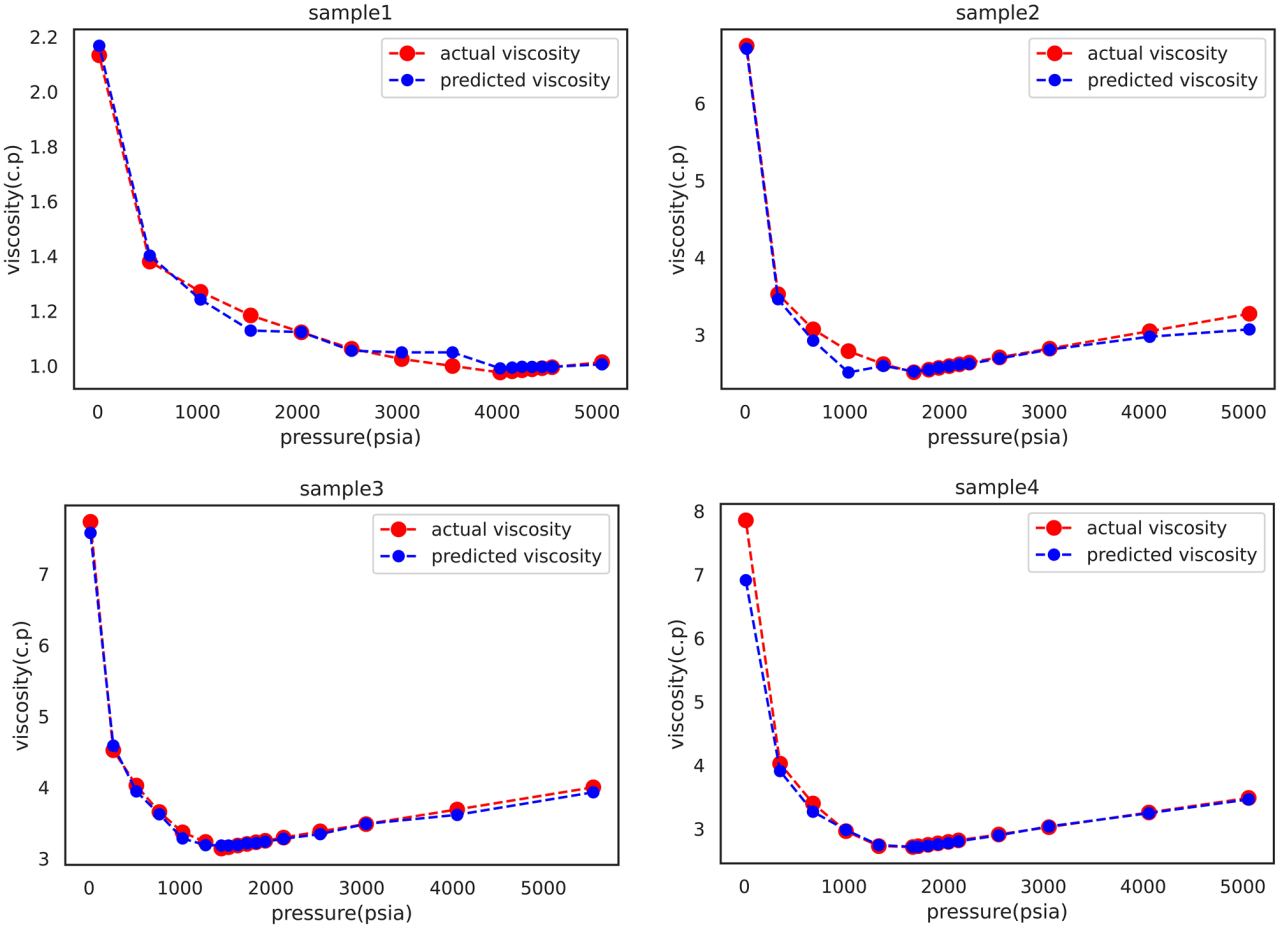
Graphical illustration for comparison between experimental viscosity values and the XGBoost model estimations of four Iranian oil samples at different pressures.

Table 6 presents the experimental viscosity values and the XGBoost model estimations for four Iranian oil samples at different pressures. Also, in order to provide a better outlook a graphical illustration is presented corresponding to each sample in Fig. 5. Hence, it can be concluded more confidently that the XGBoost model can accurately estimate viscosity regardless of the pressure range and oil type.

**Table 6:** Experimental viscosity values and the XGBoost model estimations for four Iranian oil samples at different pressures.

P (psia)	Real vis (c.p)	Model vis (c.p)	P (psia)	Real vis (c.p)	Model vis (c.p)
*Sample 1*			*Sample 2*		
5050	1.013337	1.005351	5058	3.271139	3.068417
4550	0.994974	0.995953	4057	3.045688	2.973398
4449	0.991199	0.995935	3054	2.819787	2.806714
4349	0.987460	0.995935	2551	2.706499	2.691341
4249	0.983736	0.996364	2248	2.638256	2.621846
4149	0.980025	0.993490	2148	2.615733	2.612640
**4030**	0.975609	0.990494	2046	2.592760	2.591791
3553	0.999333	1.048907	1942	2.569337	2.573254
3045	1.024600	1.048801	1842	2.546814	2.545099
2542	1.062203	1.054982	**1692.4**	2.513119	2.523687
2039	1.122385	1.122989	1386	2.618059	2.595768
1532	1.183673	1.128482	1036	2.786707	2.510240
1030	1.269898	1.241801	682	3.069599	2.923910
521	1.380175	1.402066	330	3.525551	3.460288
14.7	2.132610	2.167524	14.7	6.746075	6.707733
*Sample 3*			*Sample 4*		
5548	4.000876	3.931077	5058	3.484792	3.461583
4050	3.689539	3.614249	4057	3.255271	3.244958
3047	3.484213	3.486671	3054	3.025292	3.036069
2543	3.381709	3.343235	2551	2.909958	2.895391
2140	3.294197	3.277449	2148	2.817554	2.802215
1938	3.250343	3.236980	2046	2.794166	2.774242
1837	3.228422	3.217844	1942	2.770319	2.754894
1736	3.205814	3.212879	1842	2.747390	2.733122
1635	3.182533	3.188726	1743	2.724690	2.715401
1534	3.159252	3.183109	**1688.8**	2.712198	2.714444
**1454.4**	3.140811	3.182876	1348	2.727040	2.742313
1281	3.233294	3.188648	1019	2.962749	2.980694
1028	3.369125	3.282739	688	3.398542	3.267978
773	3.654729	3.621993	358	4.025803	3.907511
520	4.026668	3.94458	14.7	7.854152	6.910652
266	4.522285	4.587628			
14.7	7.734326	7.578169			

Bubble point pressure values are shown in [bold].

## Conclusion

In this study, GBDT based machine learning algorithms, including GradientBoosting, CatBoost, and XGBoot were adapted in order to predict oil viscosity in the reservoir as a function of pressure with the black oil approach. The results showed that the XGBoost model is relatively superior to other methods (CatBoost and GradientBoosting). The following two conclusions can be inferred:

1. Compared to the black oil approach employing the LSSVM model, the results showed that the XGBoost model provided a significant 10% error reduction in the saturated region.
2. Compared to the compositional approach employing the DTs model, the results showed that despite using 21 input parameters, the XGBoost model provided a 1.4% error reduction with only four input parameters and no need for oil composition information.

The following points can be presented to complete the aforementioned discussion:

1. The XGBoost algorithm is a relatively new GBDT based method. In this algorithm, trees of equal depths are created consecutively. An advantage of this model is the much shorter runtime than those of other GBDT based models in all computational environments due to the use of parallel processing.
2. Another important advantage of this model is that it avoids retaining the training data, which prevents overfitting. It is also due to the use of L1 and L2 regularization.
  L1 regularization prevents the overfitting of the model by shrinking the parameters towards 0. This can remove the effect of some features.
  L2 regularization prevents the overfitting of the model by making weights to be small, but not forcing them to be absolutely 0.
3. this model can also handle NaN or missing data values.

## Supplementary Information

Supplementary Information.

## Data Availability

The data will be available upon request. The corresponding author (MRK) should be contacted for this purpose.

## Abbreviations

a: Representative of a weak learner
AARD: Average absolute relative deviation, %
AI: Artificial intelligence
ANN: Artificial neural network
DT: Decision tree
EOR: Enhanced oil recovery
F(x): Objective function
GA: Genetic algorithm
GBDT: Gradient boosting decision tree
GMDH: Group method of data handling
GOR (R_s_): Solution gas–oil ratio, SCF/STB
GPR: Gaussian process regression
$h\left( {x_{i} ;a} \right)$: Desired regression tree function
k: Number of subsets
L1: Overfitting preventer regularization
L2: Overfitting preventer regularization
LSSVM: Least squares support vector machine
$$L_{y,x} \left( {y,F\left( x \right)} \right)$$: Cost function
ML: Machine learning
N: Number of data points
NaN: Not a Number
$O_{iexp}$: Experimental/actual output
$O_{ipred}$: Predicted/estimated output
$\overline{O}$: Mean of outputs
P: Pressure, psia
PVT: Pressure–volume–temprature
R^2^: Coefficient of determination
RET: Regression ensembles tree
RF: Random forest
RMSE: Root mean square error, unit of the original value
SMLR: Supervised machine learning regression
Std: Standard deviation, unit of the original value
vis: Viscosity, cp/pa.s
x: Features of interest
XGBoost: EXtreme gradient boosting
y: Target data
