# Supplementary material for: Enhanced intelligent approach for determination of crude oil viscosity at reservoir conditions
Source: Sci Rep. 2023 Jan 30;13:1666. doi: 10.1038/s41598-023-28770-2 (PMC9887002; doi:10.1038/s41598-023-28770-2)
Supplement: Supplementary file 1 — Supplementary Information. [file 41598_2023_28770_MOESM1_ESM.pdf]

Enhanced intelligent approach for determination of crude oil viscosity at reservoir conditions

Kiana Peiro Ahmady Langeroudy [1], Parsa Kharazi Esfahani [1], Mohammad Reza Khorsand Movaghar [1,4]

[1] Department of Petroleum Engineering, Amirkabir University of Technology (Tehran Polytechnic), Box 15875-4413, 424 Hafez Avenue, Tehran, 1591634311, Iran

[*] Corresponding author: Email: m.khorsand@aut.ac.ir, Tel.: +98 21 64545133

1st author email: kiana.peiro80@aut.ac.ir, 2nd author email: pkharazi1080@aut.ac.ir

Supplementary File

1. Ensemble Classifier

An ensemble classifier is a method that uses or combines multiple classifiers to improve robustness as well as to achieve an improved classification performance from any of the constituent classifiers. Furthermore, this technique is more resilient to noise compared to the use of a single classifier. This method uses a 'divide and conquers approach where a complex problem is decomposed into multiple sub-problems that are easier to understand and solve. Ensemble approaches Schapire [1], Syarif [2] have the advantage that they can be made to adapt to any changes in the monitored data stream more accurately than single model techniques. An ensemble classifier has better accuracy than single classification techniques. The success of the ensemble approach depends on the diversity in the individual classifiers with respect to misclassified instances Lee [3]. According to Polikar [4], there are four ways to achieve this diversity, the first is to use different training data to train single classifiers, the second is to use different training parameters, the third is to use different features to train the classifiers and the final one is to combine different types of the classifier. Dietterich [5] reported that there are three main reasons why an ensemble classifier is usually significantly better than a single classifier. Firstly, the training data does not always provide sufficient information for selecting a single accurate hypothesis. Secondly, the learning processes of the weak classifier might be imperfect, and thirdly, the hypothesis space being searched might not contain the true target function while an ensemble classifier can provide a good approximation. It is well known in the data mining literature that the appropriate combination of a number of weak classifiers can yield a highly accurate global classifier Gudadhe [6]. Hence, three different ensemble classifier techniques, called bagging, boosting, and stacking, using various

weak classifiers, such as the nearest neighbor, decision tree, rule induction, and naïve Bayes Syarif [2], Cup [7], Tavallaee [8]; are introduced here.

1.1. Bagging

Bagging, which means bootstrap aggregation, is one of the simplest but most successful ensemble methods for improving unstable classification problems. For example, weak classifiers, such as decision tree algorithms, can be unstable, especially when the position of a training point changes slightly and can lead to a very different tree. This method is usually applied to decision tree algorithms, but it also can be used with other classification algorithms such as naïve Bayes, nearest neighbor rule induction, etc. The bagging technique is very useful for large and high-dimensional data, such as intrusion datasets Syarif [2], where finding a good model or classifier that can work in one step is impossible because of the complexity and scale of the problem. Bagging was first introduced by Breiman [9] to reduce the variance of a predictor. It uses multiple versions of a training set which is generated by a random draw with the replacement of N examples where N is the size of the original training set. Each of these data sets is used to train a different model. The outputs of the models are combined by voting to create a single output. Details of the bagging algorithm and its pseudo-code were given in Zhou [10].

- Random Forest: The random forest classifier consists of a combination of tree classifiers where each classifier is generated using a random vector sampled independently from the input vector, and each tree casts a unit vote for the most popular class to classify an input vector Breiman [11].
- Extra Tree: The Extra-Trees algorithm builds an ensemble of the unpruned decision or regression trees according to the classical top-down procedure Geurts [12].

1.2. Boosting

Boosting, which was introduced by Bartlett [13], is an ensemble method for boosting the performance of a set of weak classifiers into a strong classifier. This technique can be viewed as a model averaging method and it was originally designed for classification, but it can also be applied to regression. Boosting provides sequential learning of the predictors. The first one learns from the whole data set, while the following learn from training sets based on the performance of the previous one. The misclassified examples are marked and their weights are increased so they will have a higher probability of appearing in the training set of the next

predictor. It results in different machines being specialized in predicting different areas of the dataset Graczyk [14].

Dietterich [15] established that boosting is more accurate than bagging.

Some boosting methods are AdaBoost, CatBoost, GBM, and XGBoost.

- GBM: The gradient boosting algorithm fits weak learners to loss function and each weak learner model aims to correct errors made by previous weak learner models. This can strengthen the prediction performance and reduce the prediction error of the model.
- Adaboost: AdaBoost algorithm is one of the most widely used boosting techniques for constructing a strong classifier as a linear combination of weak classifiers Syarif [2].
- Catboost: CatBoost is a new gradient boosting decision tree (GBDT) algorithm that can handle categorical features well.
- XGBoost: The extreme gradient boosting algorithm (XGBoost) is a supervised gradient boosting-based ensemble learning algorithm proposed by Chen [16]. The goal of this algorithm is to create a K regression tree to obtain the predicted value of the tree group as close to the true value as possible and achieve the greatest generalization ability.

1.3. Stacking

Stacking or stacked generalization is a different technique of combining multiple classifiers. Unlike bagging and boosting, stacking is usually used to combine various different classifiers, e.g. decision tree, neural network, rule induction, naïve Bayes, logistic regression, etc. Stacking consists of two levels which are base learner as level-0 and stacking model learner as level-1. Base learner (level-0) uses many different models to learn from a dataset. The outputs of each of the models are collected to create a new dataset. In the new dataset, each instance is related to the real value that it is supposed to predict. Then that dataset is used by the stacking model learner (level-1) to provide the final output Graczyk [14]. For example, the predicted classifications from the three base classifiers, naïve bayeBayescision tree, and rule induction can be used as input variables into the nearest neighbor classifier as a stacking model learner, which will attempt to learn from the data how to combine the predictions from the different models to achieve the best classification accuracy Zhou [10].

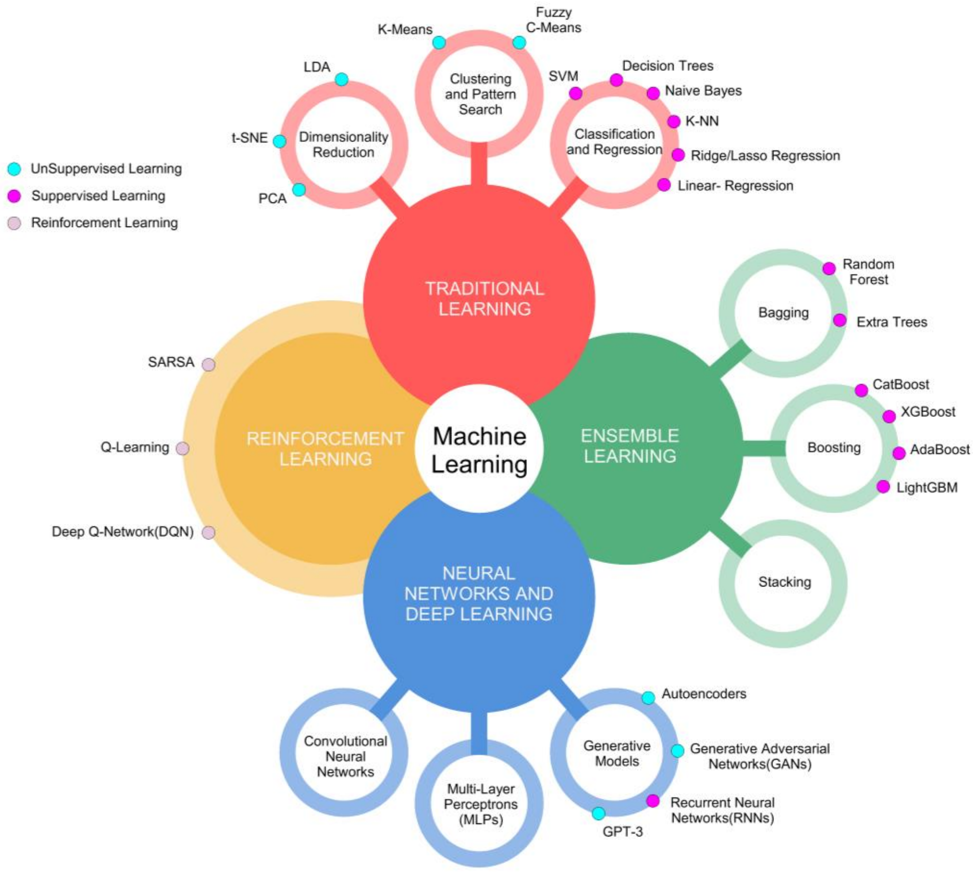

Supplementary figure S1. examples of various machine learning approaches and algorithms Karthikeyan **[17]**.

2. Model

In the present study, the ensemble type of machine learning method, an emerging line of research, is employed. An ensemble classifier integrates multiple classifiers to increase robustness and represent an improved version of classification performance from any of the constituent classifiers. Additionally, this technique, in comparison to a single classifier technique, is more resilient to noise [18]. The following ensemble methods are used in this study: GradientBoosting, CatBoost, and XGBoost machines that all these methods are developed using a gradient boosting decision tree [19,20].

2.1. GradientBoosting [21]

The boosting technique focuses on iteration and reconsideration of the errors in each step to develop a strong learner by integrating multiple weak learners. The data selected to train the model can be defined as below by assuming $x = \{x_1, x_2, \dots, x_n\}$ as the features of interest and y as the target data: $\{(x_i, y_i)\}$ $for\ i = 1, 2, \dots, n\ with\ x_i \in R^n\ and\ y_i \in R$ .In general, this method aims to find the approximate value of $\tilde{F}(x)$ for *F(x)* according to this conditions:

$$\tilde{F}(x) = arg \min_{F(x)} L_{y,x}\big(y, F(x)\big) \tag{1}$$

Where, $L_{y,x}\big(y, F(x)\big)$ is the cost function and $\arg\min_{F(x)} L_{y,x}\big(y, F(x)\big)$ is the value of *F(x)* for which $L_{y,x}\big(y, F(x)\big)$ achieves its minimum. The cost function improves the parameter prediction accuracy by reaching the smallest value. It is defined as the squared error: $L\big(y, F(x)\big) = \big(y - F(x)\big)^2$ In any case, there will always be an unavoidable error when an objective function (i.e., F(x)) is being estimated. This error can be either low or high based on the efficiency of the developed model.

Each of the weak learners tries to improve and reduce the previous weak learner's error. The GradientBoosting algorithm adapts weak learners to the cost function as much as possible in order to increase the model accuracy and decrease errors. In the first step of the GradientBoosting algorithm, a base learner is defined as $F_0(x)$, which is often considered a fixed function for simplicity. In the next step, this algorithm benefits from gradient descent to minimize the predefined cost function. The gradient descent takes proportionate steps with negative slopes in the cost function to make the result reach the local minimum.

The cost function slope is obtained from **Equation *(2)***:

$$\tilde{y}_i = -\left[\frac{\partial L\big(y_i, F(x_i)\big)}{\partial F(x_i)}\right]_{F(x) = F_{m-1}(x)}, i = 1,2, \dots, n. \tag{2}$$

If the regression tree function (i.e., $h(x_i; a)$) is used for parameter $\underline{a}$ representing a weak learner, the slope calculation range can be developed and generalized. In this function, x_i is defined as the input parameter, whereas $\underline{a}$ is defined as the parameter that should be determined **[22]**.

The desired tree is obtained by solving **Equation *(3)***:

$$a_m = arg\min_{a,\beta} \sum_{i=1}^{n} [\tilde{y}_i - \beta h(x_i; a)]^2 \quad (3)$$

Where β denotes the weighted coefficient (expansion of each weak learner's coefficients) and a_m is the parameter obtained from the mth iteration, respectively. Each decision tree is matched and adapted to its determined slope. After ρ_m (the optimal length) is determined, $F_m(x)$ is updated in the final step based on each iteration. The GradientBoosting algorithm is formalized by Algorithm 1 **[22]**.

Supplementary Algorithm S1: GradientBoosting

1. $F_0(x) = arg\min_{\rho} \sum_{i=1}^{n} L(y_i, \rho)$

2. For m = 1 to M do;

3. $\tilde{y}_i = -\left[\frac{\partial L(y_i, F(x_i))}{\partial F(x_i)}\right]_{F(x)=F_{m-1}(x)} \quad , i = 1, \ldots, n$

4. $a_m = arg\min_{a,\beta} \sum_{i=1}^{n} [\tilde{y}_i - \beta h(x_i; a)]^2$

5. $\rho_m = arg\min_{\rho} \sum_{i=1}^{n} L\big(y_i - F_{m-1}(x_i) + \rho h(x_i; a_m)\big)$

6. $F_m(x) = F_{m-1}(x) + \rho_m h(x; a_m)$

7. End for

End algorithm

2.2. CatBoost [23,24]

CatBoost is a relatively novel GBDT based method that demonstrated satisfactory results in Kaggle competitions. It also yielded considerable precisions in those competitions. A feature of GBDT is that it operates properly on datasets with numerical features. However, some

datasets may include string features (e.g., gender or country) rather than merely numerical features. Hence, these features might be of great importance and have substantial effects on the accuracy of our final prediction, it is impossible to ignore or remove them. Therefore, it is customary to convert categorical (string) features into numerical features before a dataset is trained. Unlike some other GBDT based methods, an outstanding advantage of the CatBoost model is that it can handle categorical features in the training process.

As defined earlier, categorical features are non-numerical. So, for using them in our model, we must first convert them into numbers and then begin the training process of the model. One-Hot-Encoding is one of the methods for converting categorical features into numerical ones. This technique is applicable in both preprocessing and training. However, encoding in training data points will yield far better results. It is essential to note that CatBoost utilizes this method for optimal performance.

There are other ways to handle categorical features. Another method is presented below:

First, a dataset is constructed as **Equation** ***(4)***:

$$D = \{(X_i, Y_i)\}_{i=1,\dots,n} \begin{cases} Xi = (x_{i,1}, \dots, x_{i,m}) & m \text{ is the number of features} \\ Y_i \in R & \text{value of the target} \end{cases} \quad (4)$$

n is the number of data points

To eliminate a categorical feature, we substitute it with the average target value derived from the entire training data.

Accordingly, **Equation** ***(5)*** is used instead of $x_{i,k}$.

$$\frac{\sum_{j=1}^{n} [x_{j,k} = x_{i\cdot k}] . Y_j}{\sum_{j=1}^{n} [x_{j,k} = x_{i\cdot k}]} \qquad [x_{j,k} = x_{i\cdot k}] = \begin{cases} 1 & if \quad x_{j,k} = x_{i\cdot k} \\ 0 & if \quad x_{j,k} \neq x_{i\cdot k} \end{cases} \quad (5)$$

where [·] denotes Iverson brackets

One of the problems with this solution is that it may overfit the model. To solve this problem, an alternative solution was proposed. For this purpose, the generated dataset (i.e., D) is divided into two sections: 1. training and 2. calculating the statistics.

Although this method handles the problem of overfitting to some extent, it reduces the quantity of data for training the model. Evidently, the availability of more data points can lead to better training in the model. In fact, the model can observe and analyze various data in this case.

CatBoost is designed to reduce the overfitting of the model. Unlike the aforementioned solution, it does not divide the data and uses the entire data to train the model. In other words,

we apply a random permutation of the dataset and then we calculate the mean of the target value for each example with the same category value placed before the given one in the permutation.

Consider that $\sigma = (\sigma_1, \dots, \sigma_n)$ is the permutation, we substitute $x_{\sigma_p,k}$ with

$$\frac{\sum_{j=1}^{p-1}\left[x_{\sigma_{j,k}}=x_{\sigma_{p,k}}\right]\cdot y_{\sigma j}+\beta\cdot p}{\sum_{j=1}^{p-1}\left[x_{\sigma_{j,k}}=x_{\sigma_{p\cdot k}}\right]+\beta} \quad (6)$$

P: prior value β: weight of the prior

It is worthy to be noted that, using the prior value diminishes the effect of data noise.

Another challenge that arises while converting categorical features into numerical ones would be the possible change in the primary distribution of the features. The problem is that it causes deviation in the solution. CatBoost offers a solution, which is described ahead.

Theoretical analysis to overcome the gradient bias called ordered boosting, was proposed by **[25]**. The pseudo-code of ordered boosting is expressed in Algorithm 2:

Supplementary Algorithm S2: Ordered boosting

Input: $\{(X_k, Y_k)\}_{k=1}^{n}$ *ordered according to* σ, *the number of trees* I ;

σ ←*random permutation of [1, n]*

M_i ← *0 for i = 1, …, n*

$for\ t \leftarrow 1\ to\ I\ do$

  $for\ i \leftarrow 1\ to\ n\ do$

    $r_i \leftarrow y_i - M_{\sigma(i)-1}(X_i);$

    $for\ i \leftarrow 1\ to\ n\ do$

      $\Delta M \leftarrow Learn\ Model\left[\left(X_i r_j\right): \sigma(j) \leq i\right]$

      $M_i \rightarrow M_i + \Delta M$

Return M_n

Please refer to the mentioned paper [24] for further information regarding this algorithm and its parameters.

2.3. XGBoost [26]

The extreme gradient boosting (XGBoost) algorithm, designed and introduced by Chen, et al.[27], is among the modern machine learning methods based on the gradient boosting decision tree. This algorithm aims to approximate the estimated value to the real value as much as possible by creating a large number of trees (e.g., *k*) in order to minimize errors and maximize adaptability. This algorithm integrates weak learners to create a strong learner. However, weak learners are created through residual fitting in this algorithm [28,29]. XGBoost model extends the cost function of the first-order Taylor and presents the second-order derivative information to make the model converge faster when the model is learning. Due to adding a regularization section to the cost function, the XGBoost algorithm prevents complexity and reduces the risk of overfitting.

The general process of the XGBoost algorithm is as follows:

Regard $D = \{(x_i, y_i)\}$ as a dataset with *n* samples and *d* features in each sample. y_i represents the target parameter of sample i. Then, a classification and regression tree (CART) is used as a base model. In the next step, the XGBoost algorithm integrates and mixes *k* base models to estimate the final solution.

$$\hat{y}_i = \sum_{k=1}^{K} f_k\,(x_i) \tag{7}$$

where $f_k(0)$ is the expression of tree *k* and *k* is the number of trees.

As discussed earlier, regularization is added to the cost function to improve the model performance and reduce its complexity. The regularization section and the cost function will create the XGBoost objective function as mentioned in **Equation *(8)***:

$$obj^{(k)} = \sum_{i=1}^{n} l\left[y_i, \hat{y}_i^{(1,-1)} + f_t(x_i)\right] + \sum_{k} \Omega(f_k) \tag{8}$$

$$\Omega(f) = \gamma T + \frac{1}{2}\lambda\|w\|^2 \tag{9}$$

Supplementary table S1 introduces all parameters used in the **Equation *(8)*** and **Equation *(9)*** briefly.

Supplementary table S1: list of all parameters used in ***Equation* (8)** and ***Equation* (9)**

$\hat{y}_i^{(k-1)}$	sum of the output values of the previous (k−1) trees
y_i	real value
$\hat{y}_i$	predicted value
$f_k(x_i)$	output result of tree k
l	differentiable convex loss function
$\Omega(\cdot)$	penalty term
γ	regularization parameter of leaf weight
λ	regularization parameter of number
w	Value of the leaf node
T	Number of the leaf node

The objective function after removing the constant term becomes as **Equation *(10)***:

$$obj^{(k)} = \sum_{j=1}^{T}\left[\left(\Sigma_{i\in I_j} g_i\right) w_j + \frac{1}{2}\left(\Sigma_{i\in I_j} h_i + \lambda\right) w_j^2\right] + \gamma T \quad (10)$$

In this equation, w_j is the weight of leaf node j. In order to simplify the **Equation *(10)***, $H_i = \Sigma_{i\in I_j} h_i$, $G_i = \Sigma_{i\in I_j} g_i$ was defined and placed to achieve this equation:

$$obj^{(k)} = \sum_{j=1}^{T}\left[G_i w_j + \frac{1}{2}(H_i + \lambda) w_j^2\right] + \gamma T \quad (11)$$

In **Equation *(11)***, the leaf node w_j is an uncertain value. Therefore, the objective function $obj^{(k)}$ is calculated for the first derivative of w_j, and the optimal value w_j^* of the leaf node j is solved as:

$$w_j^* = -\frac{G_i}{H_i + \lambda} \quad (12)$$

To minimize $obj^{(k)}$, w_j^* defined as above is now placed in the equation:

$$obj^{(k)} = -\frac{1}{2}\sum_{j=1}^{T}\frac{G_i^2}{H_i + \lambda} + \gamma T \quad (13)$$

In the aforementioned model, the greedy algorithm is employed to divide the input features when a CART is created. This algorithm is then implemented in the following structure by allocating the parameter m to the maximum depth of each tree.

Supplementary Algorithm S3: Exact Greedy Algorithm for Split Finding.

Input: I, instance set of the current node

Input: d, feature dimension

$gain \leftarrow 0$

$G_i \leftarrow \Sigma_{i\in I_j} g_i$, $H_i \leftarrow \Sigma_{i\in I_j} h_i$

for k =1 to m do

$$G_L \leftarrow 0, H_L \leftarrow 0$$

for j in sorted $(I, by\ x_{jk})$ do

$$G_L \leftarrow G_L + g_j \ , \ \ H_L \leftarrow H + h_j$$

$$G_R \leftarrow G + G_L \ , \ \ H_R \leftarrow H + H_L$$

$$score \leftarrow max\left(score, \frac{G_L^2}{H_L+\lambda} + \frac{G_R^2}{H_R+\lambda} - \frac{G^2}{H+\lambda}\right)$$

end

end

Output: Split with max score

In every iteration, the above algorithm navigates the features of each node from the root node. Then, for choosing the split node we use the point that has the highest score. Splitting continues to the maximum depth of the tree and then building the residue of the next tree starts. Finally, the XGBoost model is developed by collecting and using the resultant trees simultaneously. Supplementary figure S2. demonstrates the proposed algorithm structure from a different perspective for a simpler and more tangible understanding **[30]**.

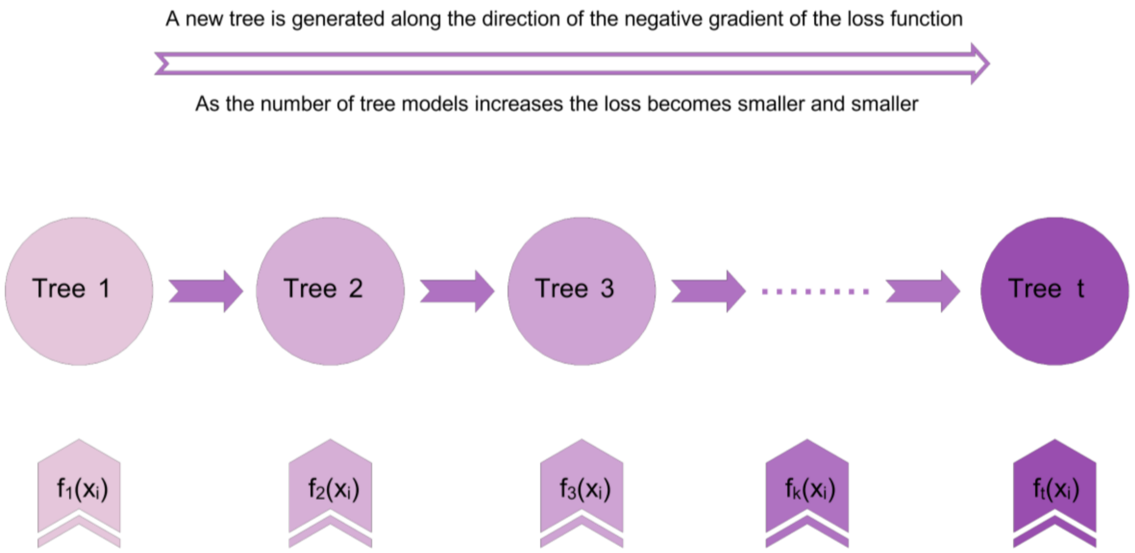

$\hat{y}_i^{(t)} = \sum_{k=1}^{T} f_k(x_i)$ where $f_k(x_i)$ represents the tree model

Supplementary figure S2. schematic of XGBoost algorithm

3. Materials and methods

A Rolling Ball viscometer (Ruska, series 1602) was considered for performing the measurements of viscosity under various pressure values. It is well-known that this equipment is more appropriate for black and volatile oils Talebkeikhah [31]. To use this equipment, a preliminary step consisting of the calibration with known viscosity standards liquid similar to the fluid to be investigated is done. The main parts of the instrument are (**1**) the stainless-steel barrel which is inclined at a defined angle and its top is closed with a plunger; and (**2**) a steel ball rolls having a diameter smaller than the bore. This latter is within the barrel, and it is fulfilled completely with the studied fluid. Due to the gravity, the ball rolls along it for a given distance. Then, the viscosity measurement is done by determining the roll time using a digital timer. Therefore, the Equation *(14)* is applied:

$$\mu_o = \alpha\left(t(\rho_{ball} - \rho_{oil})\right) + \beta \tag{14}$$

where α and β are constants which are determined from the calibration step, μ_o points out the oil viscosity, ρ_{ball} and ρ_{oil} mean the ball and oil densities, respectively, and t is the rolling time expressed in seconds. It is worth noting that the measurements are conducted at reservoir temperature and pressures varying from high values to near atmospheric pressure. It is needed to add that Rolling Ball viscometers allow a pseudo differential vaporization of gas to be performed within them, leaving the oil to fill the measuring chamber. This characteristic allows performing oil viscosity measurements in the reservoir as gas is depleted from it Talebkeikhah [31].

4. Comparison with the preexisting models

After developing the models and finding that XGBoost is the best paradigm, we have compared this latter with various available approaches for predicting the viscosity values for different circumstances (undersaturated, saturated and dead oil). The included correlations for **dead oil** are: Talebkeikhah [31], Beal [32], Beggs [33], Glaso [34], Kaye [35], Al-Khafaji [36], Egbogah [37], Petrosky [38], Labedi [39], Kartoatmodjo [40], Bennison [41], Elsharkawy [42], Hossain [43], Naseri [44], Bergman [45], Hemmati-Sarapardeh [46].For the **saturated oil**, we have compared XGBoost model with the well-known pre-existing correlations Talebkeikhah [31], Beggs [33], Al-Khafaji [36], Petrosky [38], Labedi [39], Kartoatmodjo [40], Elsharkawy [42], Hossain [43], Naseri [44], Bergman [45], Hemmati-Sarapardeh [46], Chew [47], Khan [48]. For the **undersaturated cases**, the considered preexisting models for comparison are Talebkeikhah [31], Beal [32], Petrosky [38], Labedi [39], Kartoatmodjo [40], Elsharkawy [42], Hossain [43], Hemmati-Sarapardeh [46], Khan [48], Vazquez [49], Orbey [50].

Supplementary table S2: Performance comparison with the preexisting models for dead oil.

Dead Oil

**Method**	**AARD (%)**	**RMSE**	**R^2**
Beal 1946[1]	43.038	5.769	-2.9351
Beggs and Robinson 1975[1]	28.410	3.488	-0.4382
Glaso 1980[1]	72.079	10.634	-12.3702
Kaye 1985[1]	183.635	25.854	-78.0397
Al Khafaji 1987[1]	67.148	10.549	-12.1591
Petrosky 1990[1]	81.769	10.421	-11.8404
Egbogah and Ng 1990[1]	82.717	6.684	-4.2833
Labedi 1992[1]	332.778	34.520	-139.9038
Kartoatmodjo and Schmidt 1994[1]	85.122	13.416	-20.2827
Bennison 1998[1]	66.423	5.637	-2.7578
Elsharkawy 1999[1]	93.694	7.985	-6.5397
Hossain 2005[1]	79.464	8.689	-7.9264

Naseri 2005[1]	55.457	7.001	-4.7959
Bergman 2007[1]	57.104	7.245	-5.2068
Hemmati-Sarapardeh 2013 (black oil)[1]	21.2	0.00182	0.955
Talebkeikhah [31]	6.524	0.0004	0.9921
XGBoost (this study)	8.899	0.00098	0.893

[1] reported by Talebkeikhah [31]

Supplementary table S3: Performance comparison with the preexisting models for saturated oil.

Saturated Oil

**Method**	**AARD (%)**	**RMSE**	$\mathbf{R^2}$
Chew and Connally 1[2]	33.355	1.292	0.2649
Chew and Connally 2[2]	16.056	0.457	0.9080
Chew and Connally 3[2]	15.937	0.454	0.9090
Beggs and Robinson 1975[2]	32.533	1.365	0.1805
Al Khafaji 1987[2]	20.79	1.32	0.799255
Khan 1987[2]	out of range	out of range	0
Petrosky 1990[2]	48.292	4.214	-6.8159
Labedi 1992[2]	335.686	17.352	-131.5260
Kartoatmodjo and Schmidt 1994[2]	94.530	2.083	-0.9091
Elsharkawy 1999[2]	45.654	2.682	-2.1653
Hossain 2005[2]	78.276	3.293	-3.7737
Naseri 2005[2]	57.107	3.894	-5.6731
Bergman 2007[2]	36.436	3.078	-3.1712
Hemmati-Sarapardeh 2013 (black oil)[2]	13.48	0.00038	0.979
Talebkeikhah [31]	4.485	0.00015	0.9964
XGBoost (this study)	2.870	0.00013	0.9920

[2] reported by Talebkeikhah [31]

Supplementary table S4: Performance comparison with the preexisting models for undersaturated oil.

Undersaturated Oil

**Method**	**AARD (%)**	**RMSE**	R^2
Beal 1946[3]	4.944	0.355	0.9542
Vazquez and Beggs 1980[3]	10.790	0.725	0.8086
Khan 1987[3]	3.24	0.298	0.98812
Petrosky 1990[3]	63.588	4.854	-7.5690
Labedi 1992[3]	541.263	30.469	-336.7010
Orbey and Sandler 1993[3]	5.159	0.316	0.9636
Kartoatmodjo and Schmidt 1994[3]	78.027	2.417	-1.1243
Elsharkawy 1999[3]	54.748	3.542	-3.5632
Hossain 2005[3]	362.493	6.900	-16.3194
Hemmati-Sarapardeh 2013 (black oil)[3]	1.4	0.00004	0.99
Talebkeikhah [31]	2.255	0.0001	0.9991
XGBoost (this study)	0.902	0.00005	0.9986

[3] reported by Talebkeikhah [31]

The well-known compositional paradigms for estimating crude oil viscosity are those derived from:

- The combination of corresponding state theory and the concept of residual viscosity Lohrenz [51], Al-Syabi [52]
- The corresponding state theory Pedersen [53], Aasberg-Petersen [54], Lindeloff [55]
- Cubic equations of state (EOSs) Peng [56], Guo [57]

Some of the most important compositional paradigms are listed in the following table and compared with machine learning methods. It should be noted quantities are extracted from Talebkeikhah [31].

Supplementary table S5: Performance comparison with the preexisting compositional models.

Compositional (overall)

**Method**	**AARD (%)**	**RMSE**	**R^2**
Petrol[4]	> 100	> 100	< 0
Elsharkawy[4]	34.545	1.672	0.1453
LBC[4]	92.916	1.763	0.0492
Extended LBC[4]	85.134	2.593	-1.0575
Talebkeikhah **[31]**	3.379	0.0001	0.9970
XGBoost (this study)	1.968	0.00008	0.9976

[4] reported by Talebkeikhah **[31]**

References

[1] Schapire RE. The boosting approach to machine learning: An overview. Nonlinear estimation and classification 2003:149-71.

[2] Syarif I, Zaluska E, Prugel-Bennett A, Wills G, editors. Application of bagging, boosting and stacking to intrusion detection. International Workshop on Machine Learning and Data Mining in Pattern Recognition; 2012: Springer.

[3] Lee KC, Cho H. Performance of ensemble classifier for location prediction task: emphasis on Markov Blanket perspective. International Journal of u-and e-Service, Science and Technology 2010;3:2010.

[4] Polikar R. Ensemble based systems in decision making. IEEE Circuits and systems magazine 2006;6:21-45.

[5] Dietterich TG. Machine-learning research. AI magazine 1997;18:97-.

[6] Gudadhe M, Prasad P, Wankhade LK, editors. A new data mining based network intrusion detection model. 2010 International Conference on Computer and Communication Technology (ICCCT); 2010: IEEE.

[7] Cup K. http://kdd. ics. uci. edu/databases/kddcup99/kddcup99. html. The UCI KDD Archive 1999.

[8] Tavallaee M, Bagheri E, Lu W, Ghorbani AA, editors. A detailed analysis of the KDD CUP 99 data set. 2009 IEEE symposium on computational intelligence for security and defense applications; 2009: Ieee.

[9] Breiman L. Bagging predictors. Machine learning 1996;24:123-40.

[10] Zhou Z-H. Ensemble learning, Encyclopedia of Biometrics. doi 2009;10:978-0.

[11] Breiman L. 1 RANDOM FORESTS--RANDOM FEATURES. 1999.

[12] Geurts P, Ernst D, Wehenkel L. Extremely randomized trees. Machine learning 2006;63:3-42.

[13] Bartlett P, Freund Y, Lee WS, Schapire RE. Boosting the margin: A new explanation for the effectiveness of voting methods. The annals of statistics 1998;26:1651-86.

[14] Graczyk M, Lasota T, Trawiński B, Trawiński K, editors. Comparison of bagging, boosting and stacking ensembles applied to real estate appraisal. Asian conference on intelligent information and database systems; 2010: Springer.

[15] Dietterich TG, editor Ensemble methods in machine learning. International workshop on multiple classifier systems; 2000: Springer.

[16] Chen T, Guestrin C, editors. Xgboost: A scalable tree boosting system. Proceedings of the 22nd acm sigkdd international conference on knowledge discovery and data mining; 2016.

[17] Karthikeyan A, Priyakumar U. Artificial intelligence: machine learning for chemical sciences. Journal of Chemical Sciences 2022;134:1-20

[18] Syarif I, Zaluska E, Prugel-Bennett A, Wills G, editors. Application of bagging, boosting and stacking to intrusion detection. International Workshop on Machine Learning and Data Mining in Pattern Recognition; 2012: Springer.

[19] Al Daoud E. Comparison between XGBoost, LightGBM and CatBoost using a home credit dataset. International Journal of Computer and Information Engineering 2019;13:6-10.

[20] Habib A-ZSB, Tasnim T, Billah MM, editors. A study on coronary disease prediction using boosting-based ensemble machine learning approaches. 2019 2nd International Conference on Innovation in Engineering and Technology (ICIET); 2019: IEEE.

[21] Nie P, Roccotelli M, Fanti MP, Ming Z, Li Z. Prediction of home energy consumption based on gradient boosting regression tree. Energy Reports 2021;7:1246-55.

[22] Friedman JH. Greedy function approximation: a gradient boosting machine. Annals of statistics 2001:1189-232.

[23] Dorogush AV, Ershov V, Gulin A. CatBoost: gradient boosting with categorical features support. arXiv preprint arXiv:181011363 2018.

[24] Huang G, Wu L, Ma X, Zhang W, Fan J, Yu X et al. Evaluation of CatBoost method for prediction of reference evapotranspiration in humid regions. Journal of Hydrology 2019;574:1029-41.

[25] Prokhorenkova L, Gusev G, Vorobev A, Dorogush AV, Gulin A. CatBoost: unbiased boosting with categorical features. Advances in neural information processing systems 2018;31.
[26] Liu Y, Wang H, Fei Y, Liu Y, Shen L, Zhuang Z et al. Research on the prediction of green plum acidity based on improved XGBoost. Sensors 2021;21:930.
[27] Chen T, Guestrin C, editors. Xgboost: A scalable tree boosting system. Proceedings of the 22nd acm sigkdd international conference on knowledge discovery and data mining; 2016.
[28] Xiao Z, Luo Al. XGBoost based stellar spectral classification and quantized feature. Spectroscopy and Spectral Analysis 2019;39:3292-6.
[29] Zopluoglu C. Detecting examinees with item preknowledge in large-scale testing using extreme gradient boosting (XGBoost). Educational and psychological measurement 2019;79:931-61.
[30] Mo H, Sun H, Liu J, Wei S. Developing window behavior models for residential buildings using XGBoost algorithm. Energy and Buildings 2019;205:109564.
[31] Talebkeikhah M, Amar MN, Naseri A, Humand M, Hemmati-Sarapardeh A, Dabir B et al. Experimental measurement and compositional modeling of crude oil viscosity at reservoir conditions. Journal of the Taiwan Institute of Chemical Engineers 2020;109:35-50.
[32] Beal C. The viscosity of air, water, natural gas, crude oil and its associated gases at oil field temperatures and pressures. Transactions of the AIME 1946;165:94-115.
[33] Beggs HD, Robinson JR. Estimating the viscosity of crude oil systems. Journal of Petroleum technology 1975;27:1140-1.
[34] Glaso O. Generalized pressure-volume-temperature correlations. Journal of Petroleum Technology 1980;32:785-95.
[35] Kaye S. Offshore California viscosity correlations. COFRC, TS85000940 1985.
[36] Al-Khafaji AH, Abdul-Majeed GH, Hassoon SF. Viscosity correlation for dead, live and undersaturated crude oils. J Pet Res 1987;6:1-16.
[37] Egbogah EO, Ng JT. An improved temperature-viscosity correlation for crude oil systems. Journal of Petroleum Science and Engineering 1990;4:197-200.
[38] Petrosky GE. PVT correlations for gulf of mexico crude oils: University of Southwestern Louisiana; 1990.
[39] Labedi R. Improved correlations for predicting the viscosity of light crudes. Journal of Petroleum Science and Engineering 1992;8:221-34.
[40] Kartoatmodjo T, Schmidt Z. Large data bank improves crude physical property correlations. Oil and Gas Journal;(United States) 1994;92.

[41] Bennison T, editor Prediction of heavy oil viscosity. IBC Heavy Oil Field Development Conference; 1998: Citeseer.
[42] Elsharkawy A, Alikhan A. Models for predicting the viscosity of Middle East crude oils. Fuel 1999;78:891-903.
[43] Hossain MS, Sarica C, Zhang H-Q, Rhyne L, Greenhill K, editors. Assessment and development of heavy oil viscosity correlations. SPE International Thermal Operations and Heavy Oil Symposium; 2005: OnePetro.
[44] Naseri A, Nikazar M, Dehghani SM. A correlation approach for prediction of crude oil viscosities. Journal of petroleum science and engineering 2005;47:163-74.
[45] Bergman DF, Sutton RP, editors. An update to viscosity correlations for gas-saturated crude oils. SPE annual technical conference and exhibition; 2007: OnePetro.
[46] Hemmati-Sarapardeh A, Khishvand M, Naseri A, Mohammadi AH. Toward reservoir oil viscosity correlation. Chemical Engineering Science 2013;90:53-68.
[47] Chew J-N, Connally CA. A viscosity correlation for gas-saturated crude oils. Transactions of the AIME 1959;216:23-5.
[48] Khan S, Al-Marhoun M, Duffuaa S, Abu-Khamsin S, editors. Viscosity correlations for Saudi Arabian crude oils. Middle East Oil Show; 1987: OnePetro.
[49] Vazquez M, Beggs HD, editors. Correlations for fluid physical property prediction. SPE Annual Fall Technical Conference and Exhibition; 1977: OnePetro.
[50] Orbey H, Sandler SI. The prediction of the viscosity of liquid hydrocarbons and their mixtures as a function of temperature and pressure. The Canadian Journal of Chemical Engineering 1993;71:437-46.
[51] Lohrenz J, Bray BG, Clark CR. Calculating viscosities of reservoir fluids from their compositions. Journal of Petroleum Technology 1964;16:1171-6.
[52] Al-Syabi Z, Danesh A, Tohidi B, Todd A, Tehrani D. A residual viscosity correlation for predicting the viscosity of petroleum reservoir fluids over wide ranges of pressure and temperature. Chemical engineering science 2001;56:6997-7006.
[53] Pedersen KS, Fredenslund A. An improved corresponding states model for the prediction of oil and gas viscosities and thermal conductivities. Chemical Engineering Science 1987;42:182-6.
[54] Aasberg-Petersen K, Knudsen K, Fredenslund A. Prediction of viscosities of hydrocarbon mixtures. Fluid phase equilibria 1991;70:293-308.

[55] Lindeloff N, Pedersen KS, Ronningsen HP, Milter J, editors. The corresponding states viscosity model applied to heavy oil systems. Canadian International Petroleum Conference; 2003: OnePetro.
[56] Peng D-Y, Robinson DB. A new two-constant equation of state. Industrial & Engineering Chemistry Fundamentals 1976;15:59-64.
[57] Guo X-Q, Wang L-S, Rong S-X, Guo T-M. Viscosity model based on equations of state for hydrocarbon liquids and gases. Fluid Phase Equilibria 1997;139:405-21.
